# Supplementary figures and images for: FANCJ helicase promotes DNA end resection by facilitating CtIP recruitment to DNA double-strand breaks
Source: PLoS Genet. 2020 Apr 6;16(4):e1008701. doi: 10.1371/journal.pgen.1008701 (PMC7162537; doi:10.1371/journal.pgen.1008701)

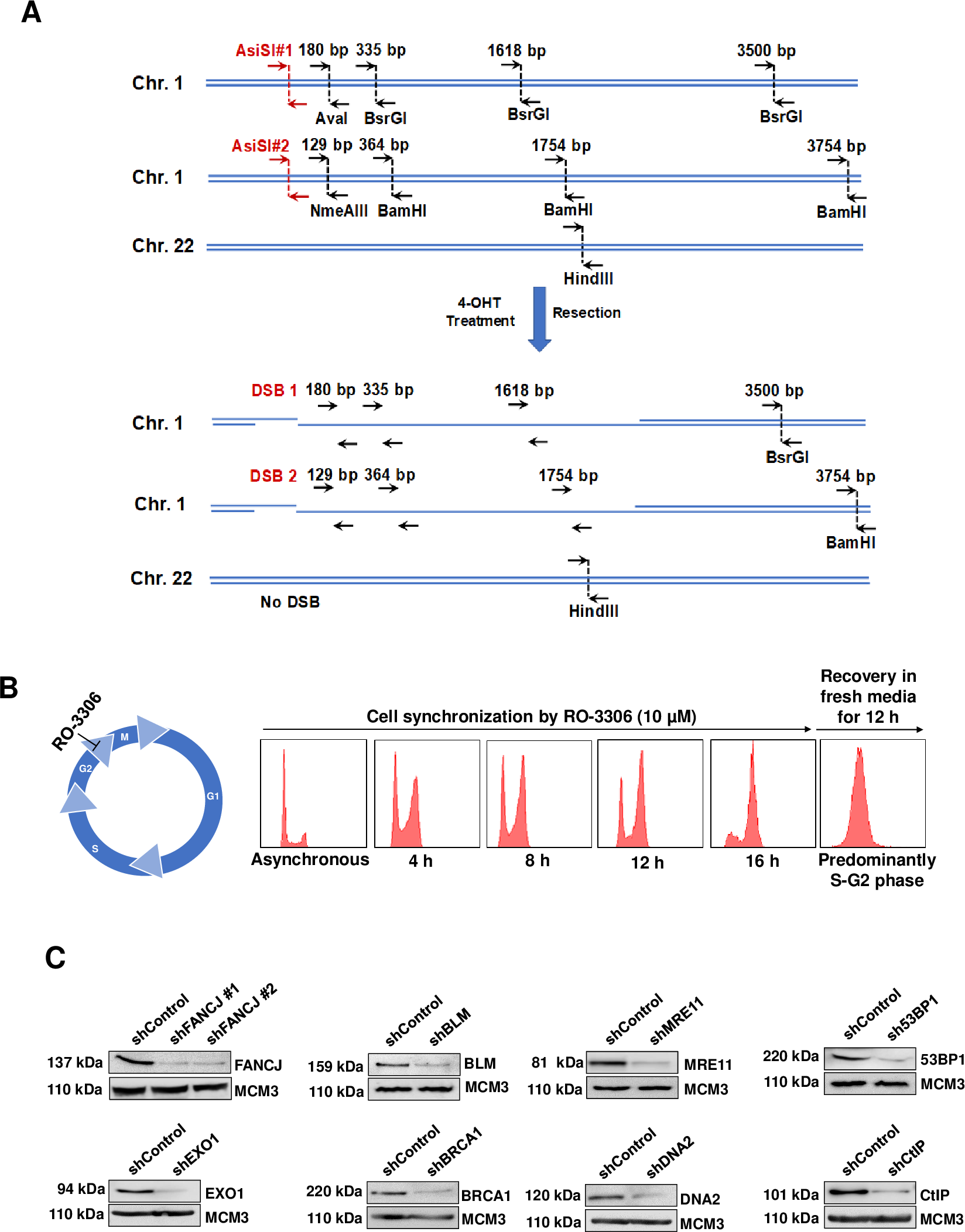

Supplement: S1 Fig — (A) Design of qPCR primers for measurement of DSB% at two AsiSI sites (red arrows: DSB1 and DSB2) located on Chromosome 1 and measurement of resection at sites adjacent to the AsiSI sites (black arrows) [24]. The primers on Chromosome 22 (‘No DSB’) were used as negative control. The primer pairs for ‘DSB1’ are across AvaI and BsrGI restriction sites; and for ‘DSB2’ are across NmeAIII and BamHI restriction sites. The primer pair for ‘No DSB’ is across a HindIII restriction site. (B) Experimental design for cell synchronisation by RO-3306 at S/G2 phase for measurement of end resection (detailed protocol in Materials and Methods section). (C) Validation of shRNA mediated knockdown of indicated proteins by immunoblotting with respective antibodies after 48 h and MCM3 as loading control. (TIF) [file pgen.1008701.s001.tif]

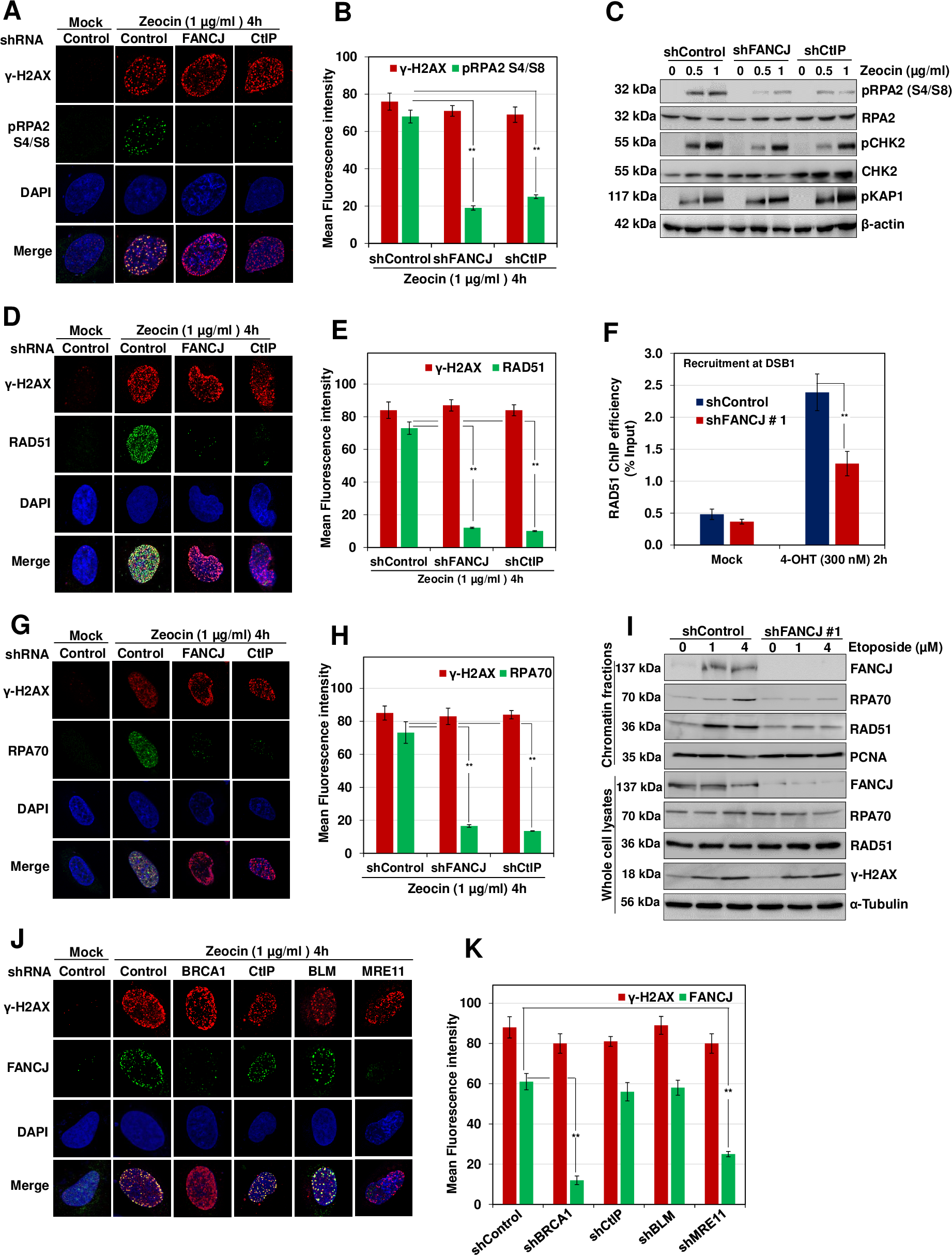

Supplement: S2 Fig — (A) ER-AsiSI U2OS cells depleted for the indicated proteins were treated with zeocin (1μg/ml) for 4 h or mock treated. Cells were fixed and stained with γ-H2AX and pRPA2 (S4/S8) antibodies to detect ssDNA generated by end resection. Representative image for γ-H2AX and pRPA2 (S4/S8) foci are shown. (B) Graph represents the mean fluorescence intensity of γ-H2AX and pRPA2 (S4/S8) foci/nucleus from indicated cells in (A). N = 3; error bars indicate standard deviation (SD) and statistical significance was measured by two-tailed Student’s t-test of unequal variance. *p < 0.05; **p < 0.01; ***p < 0.001; N.S., non-significant. (C) ER-AsiSI U2OS cells treated with either control shRNA, shFANCJ #1 or shCtIP were treated with increasing dose of zeocin (0, 0.5 and 1 μg/ml) for 4 h. Whole cell lysates were separated on 10% SDS-PAGE and probed for the indicated proteins to measure their damage induced enrichment in the cell. (D) ER-AsiSI U2OS cells depleted for the indicated proteins were treated with zeocin (1μg/ml) for 4 h or mock treated. Cells were fixed and stained with γ-H2AX and RAD51 antibodies. Representative image for γ-H2AX and RAD51 foci are shown. (E) Graph represents the mean fluorescence intensity of γ-H2AX and RAD51 foci/nucleus from indicated cells in (D). N = 3; error bars indicate standard deviation (SD) and statistical significance was measured by two-tailed Student’s t-test of unequal variance. *p < 0.05; **p < 0.01; ***p < 0.001; N.S., non-significant. (F) FANCJ depleted ER-AsiSI U2OS cells were treated with 300 nM 4-OHT for 2 h or mock treated, and ChIP assays were performed using antibody directed against RAD51. ChIP efficiencies (as percent of input immunoprecipitated) were measured by semiquantitative PCR at 80 bp from AsiSI induced DSB1 site. N = 3, with error bars indicating SD and statistical significance was measured by two-tailed Student’s t-test of unequal variance. *p < 0.05; **p < 0.01; ***p < 0.001; N.S., non-significant. (G) ER-AsiSI U2OS cells [file pgen.1008701.s002.tif]

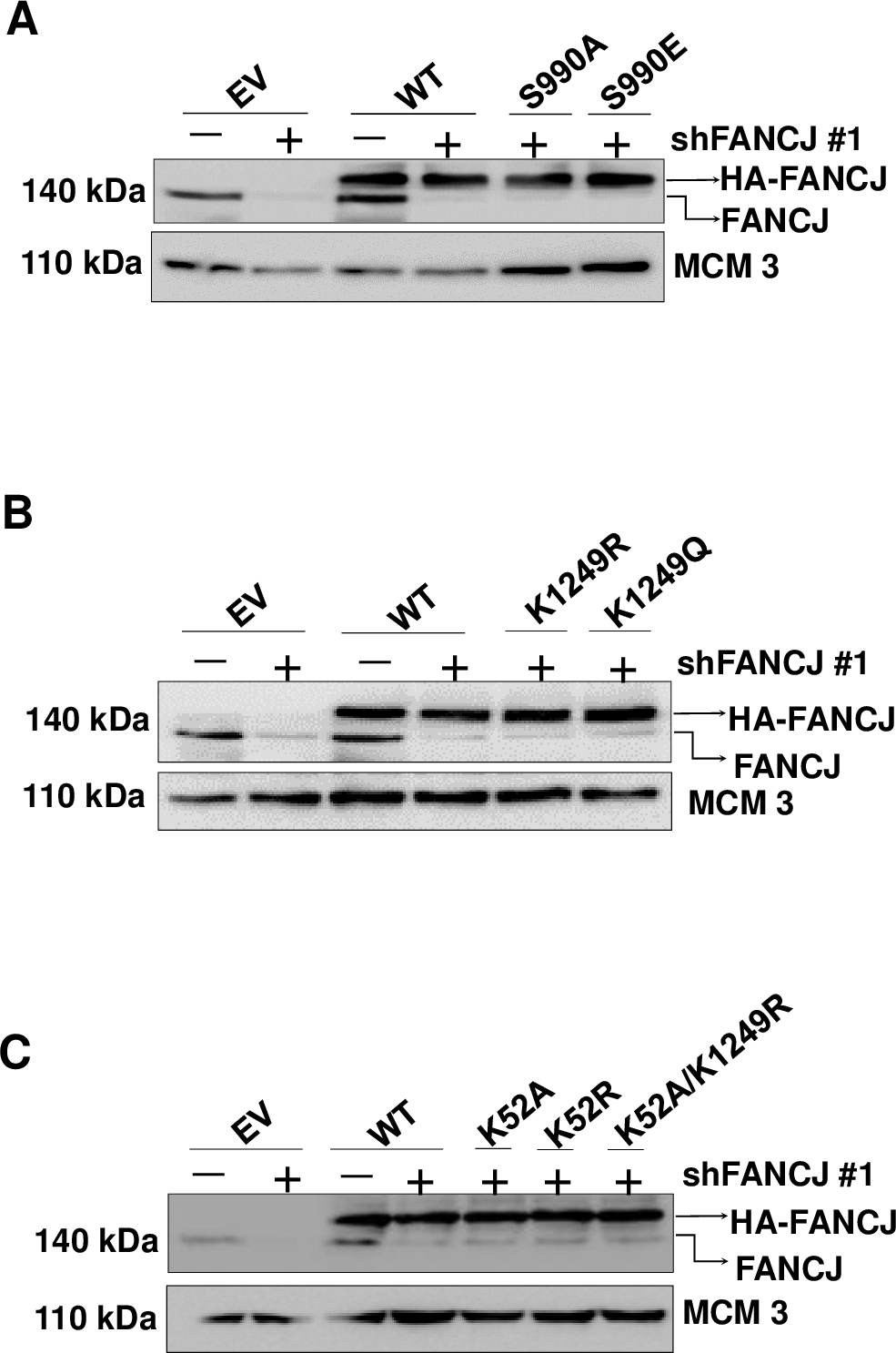

Supplement: S3 Fig — (A)Relative protein levels of endogenous FANCJ and WT/S990A/S990E-HA-6xHis-FANCJ. (B) Relative protein levels of endogenous FANCJ and WT/K1249R/K1249Q-HA-6xHis-FANCJ. (C) Relative protein levels of endogenous FANCJ and WT, K52A, K52R, K52A/1249R- HA-6xHis-FANCJ. In (A), (B) and (C), western blotting was carried out using FANCJ specific antibody. (TIF) [file pgen.1008701.s003.tif]
