# Supplementary material for: FANCJ helicase promotes DNA end resection by facilitating CtIP recruitment to DNA double-strand breaks
Source: PLoS Genet. 2020 Apr 6;16(4):e1008701. doi: 10.1371/journal.pgen.1008701 (PMC7162537; doi:10.1371/journal.pgen.1008701)
Supplement: S1 Table — (PDF) [file pgen.1008701.s004.pdf]

**Table S1: Sequences of qPCR Primers used for studying ssDNA generation**			
**Primer name**	**Distance from *Asi*SI**	**Oligo**	**Sequence (5′→3′)**
No DSB		FWD	ATTGGGTATCTGCGTCTAGTGAGG
		REV	GACTCAATTACATCCCTGCAGCT
DSB1	180 bp	FWD	TGTGGACTCAGGGAACTC
		REV	CAGTCGCATACATCCGAT
DSB1	335 bp	FWD	GAATCGGATGTATGCGACTGATC
		REV	TTCCAAAGTTATTCCAACCCGAT
DSB1	1618 bp	FWD	TGAGGAGGTGACATTAGAACTCAGA
		REV	AGGACTCACTTACACGGCCTTT
DSB1	3500 bp	FWD	TCCTAGCCAGATAATAATAGCTATACAAACA
		REV	TGAATAGACAGACAACAGATAAATGAGACA
DSB2	129 bp	FWD	GCTGGAGTGTACGGGCCC
		REV	GTTCCTGGCCGGGACAGC
DSB2	364 bp	FWD	CCAGCAGTAAAGGGGAGACAGA
		REV	CTGTTCAATCGTCTGCCCTTC
DSB2	1754 bp	FWD	GAAGCCATCCTACTCTTCTCACCT
		REV	GCTGGAGATGATGAAGCCCA
DSB2	3564 bp	FWD	GCCCAGCTAAGATCTTCCTTCA
		REV	CTCCTTTGCCCTGAGAAGTGA
Across DSB1		FWD	GATGTGGCCAGGGATTGG
		REV	CACTCAAGCCCAACCCGT
Across DSB2		FWD	GAGGAGCCTCTCCTGCAGC
		REV	GAACCAGACCTACCTCCAGGG
