# Supplementary material for: FANCJ helicase promotes DNA end resection by facilitating CtIP recruitment to DNA double-strand breaks
Source: PLoS Genet. 2020 Apr 6;16(4):e1008701. doi: 10.1371/journal.pgen.1008701 (PMC7162537; doi:10.1371/journal.pgen.1008701)
Supplement: S2 Table — (PDF) [file pgen.1008701.s005.pdf]

<table>
<tr><th colspan="5">Table S2: DSB% at DSB1 and DSB2 sites</th></tr>
<tr><th colspan="2">Figure no.</th><th>Treatment</th><th>DSB% at DSB1</th><th>DSB% at DSB2</th></tr>
<tr><td colspan="2">Fig 1J</td><td>shControl</td><td>28.5 ± 1.9</td><td>19.5 ± 3.1</td></tr>
<tr><td colspan="2"></td><td>shBRCA1</td><td>18.3 ± 2.5</td><td>7.95 ± 2.3</td></tr>
<tr><td colspan="2"></td><td>shMRE11</td><td>19.7 ± 5.03</td><td>7.57 ± 2.7</td></tr>
<tr><td colspan="2"></td><td>shCtIP</td><td>19.5 ± 4.3</td><td>8.47 ± 2.3</td></tr>
<tr><td colspan="2"></td><td>shFANCJ # 1</td><td>23.5 ± 2.88</td><td>15.7 ± 4.24</td></tr>
<tr><td colspan="2"></td><td>shFANCJ # 2</td><td>23.3 ± 5.05</td><td>16.5 ± 2.1</td></tr>
<tr><td colspan="2"></td><td>shDNA2</td><td>23.7 ± 4.76</td><td>16.8 ± 3.7</td></tr>
<tr><td colspan="2"></td><td>sh53BP1</td><td>25.3 ±4.5</td><td>17.9 ± 2.28</td></tr>
<tr><td colspan="2">Fig. 2G</td><td>shControl</td><td>22.3 ± 5.3</td><td>15.8 ± 2.01</td></tr>
<tr><td colspan="2"></td><td>shFANCJ # 1</td><td>26.7 ± 3.05</td><td>17.3 ± 3.07</td></tr>
<tr><td colspan="2"></td><td>shCtIP</td><td>27.7 ± 2.08</td><td>17.9 ± 3.19</td></tr>
<tr><td colspan="2"></td><td>shFANCJ # 1+ shCtIP</td><td>21.6 ± 7.6</td><td>12.22 ± 1.03</td></tr>
<tr><td colspan="2">Fig. 3D</td><td>shControl</td><td>24.3 ± 3.05</td><td>13.7 ± 1.16</td></tr>
<tr><td colspan="2"></td><td>shFANCJ # 1</td><td>16.7 ± 0.57</td><td>9.45 ± 0.79</td></tr>
<tr><td colspan="2"></td><td>shFANCJ # 1 + WT-FANCJ</td><td>18.3 ± 5.77</td><td>10.4 ± 0.87</td></tr>
<tr><td colspan="2"></td><td>shFANCJ # 1 + 1-881 FANCJ</td><td>26.7 ± 3.5</td><td>15.11 ± 1.3</td></tr>
<tr><td colspan="2">Fig. 4C</td><td>shControl</td><td>22.7 ± 6.6</td><td>12.8 ± 1.08</td></tr>
<tr><td colspan="2"></td><td>shFANCJ # 1</td><td>20.0 ± 4.58</td><td>11.3 ± 0.95</td></tr>
<tr><td colspan="2"></td><td>shFANCJ # 1 + WT-FANCJ</td><td>21.0 ± 1.26</td><td>11.8 ± 1.00</td></tr>
<tr><td colspan="2"></td><td>shFANCJ # 1 + S990A-FANCJ</td><td>24.3 ± 2.52</td><td>13.8 ± 1.16</td></tr>
<tr><td colspan="2"></td><td>shFANCJ # 1 + S990E-FANCJ</td><td>28.5 ± 1.28</td><td>16.13 ± 1.36</td></tr>
<tr><td colspan="2">Fig. 5C</td><td>shControl</td><td>19.7 ± 5.5</td><td>11.15 ± 0.94</td></tr>
<tr><td colspan="2"></td><td>shFANCJ # 1</td><td>19.3 ± 0.6</td><td>10.95 ± 0.92</td></tr>
<tr><td colspan="2"></td><td>shFANCJ # 1 + WT-FANCJ</td><td>21.0 ± 1.8</td><td>11.9 ± 1.00</td></tr>
<tr><td colspan="2"></td><td>shFANCJ # 1 + K1249R-FANCJ</td><td>19.3 ± 3.78</td><td>15.01 ± 2.84</td></tr>
<tr><td colspan="2"></td><td>shFANCJ # 1 + K1249Q-FANCJ</td><td>23.6 ± 4.6</td><td>18.3 ± 3.46</td></tr>
<tr><td rowspan="8">Fig. 8C</td><td rowspan="4">shControl</td><td>shCtIP + EV</td><td>22.0 ± 2.2</td><td rowspan="8"></td></tr>
<tr><td>shCtIP + WT-CtIP (R)</td><td>21.4 ± 3.7</td></tr>
<tr><td>shCtIP + S327A-CtIP (R)</td><td>24.0 ± 1.9</td></tr>
<tr><td>shCtIP + T847A-CtIP (R)</td><td>19.8 ± 5.6</td></tr>
<tr><td rowspan="4">shFANCJ</td><td>shCtIP + EV</td><td>18.5 ± 6.2</td></tr>
<tr><td>shCtIP + WT-CtIP (R)</td><td>21.0 ± 3.1</td></tr>
<tr><td>shCtIP + S327A-CtIP (R)</td><td>17.6 ± 4.2</td></tr>
<tr><td>shCtIP + T847A-CtIP (R)</td><td>15.6.0 ± 3.2</td></tr>
<tr><td colspan="2">Fig.8D</td><td>shControl</td><td>20.7 ± 1.43</td><td>9.54 ± 0.81</td></tr>
<tr><td colspan="2"></td><td>shFANCJ # 1</td><td>15.1 ± 0.62</td><td>10.14 ± 1.13</td></tr>
<tr><td colspan="2"></td><td>shBRCA1</td><td>17.3 ± 1.5</td><td>11.37 ± 2.71</td></tr>
<tr><td colspan="2"></td><td>shFANCJ # 1+ shBRCA1</td><td>18.5 ± 3.3</td><td>7 ± 0.68</td></tr>
<tr><td colspan="2">Fig. 9E</td><td>shControl</td><td>20.0 ± 5.2</td><td>9.04 ± 1.89</td></tr>
<tr><td colspan="2"></td><td>shFANCJ # 1</td><td>21.3 ± 3.78</td><td>9.5 ± 1.95</td></tr>
</table>

| Figure no. | Treatment | DSB% at DSB1 | DSB% at DSB2 |
|---|---|---|---|
| | shFANCJ # 1 + WT-FANCJ | 23.3 ± 4.7 | 10.5 ± 2.17 |
| | shFANCJ # 1 + K52A-FANCJ | 28.3 ± 2.08 | 12.8 ± 2.6 |
| | shFANCJ # 1 + K52R-FANCJ | 20.6 ± 1.38 | 9.3 ± 1.9 |

∗ **Summary of the % DSBs at the two selected AsiSI sites after 4 h of 4-OHT treatment**.

% DSB values were measured by qPCR using undigested gDNA samples and two sets of primers across the two AsiSI sites (Fig. 1A). The ‘No DSB’ primers were used to normalize the amount of gDNA in the qPCR reaction. DSB percentages at DSB1 and DSB2 sites in mock-treated cells were both set to zero.
