# Supplementary material for: FANCJ helicase promotes DNA end resection by facilitating CtIP recruitment to DNA double-strand breaks
Source: PLoS Genet. 2020 Apr 6;16(4):e1008701. doi: 10.1371/journal.pgen.1008701 (PMC7162537; doi:10.1371/journal.pgen.1008701)
Supplement: S3 Table — (PDF) [file pgen.1008701.s006.pdf]

Table S3: Sequences of primers used in this study

Primer name	Oligo	Sequence (5′→3′)
WT-FANCJ	FWD	ATAGAGATATCATGTCTTCAATGTGGTCT
	REV	ATAGACTCGAGTCAGTGATGGTGGTGATGGTGTGCATAGTCGGGGACGTCA TAGGGGTACTTAAAACCAGGAAA
S990A-FANCJ	FWD	TCCAGATCCACA**GCC**CCAACTTTCAAC
	REV	GTTGAAAGTTGG**GGC**TGTGGATCTGGA
S990E-FANCJ	FWD	TCCAGATCCACA**GAG**CCAACTTTCAAC
	REV	GTTGAAAGTTGG**CTC**TGTGGATCTGGA
K52A-FANCJ	FWD	ACAGGAAGTGGA**GCA**AGCTTAGCCTTA
	REV	TAAGGCTAAGCT**TGC**TCCACTTCCTGT
K52R-FANCJ	FWD	ACAGGAAGTGGA**CGA**AGCTTAGCCTTA
	REV	TAAGGCTAAGCT**TCG**TCCACTTCCTGT
K1249R-FANCJ	FWD	TTTCCTGGTTTT**AGG**TACCCCTATGAC
	REV	GTCATAGGGGTA**CCT**AAAACCAGGAAA
K1249Q FANCJ	FWD	TTTCCTGGTTTT**CAG**TACCCCTATGAC
	REV	GTCATAGGGGTA**CTG**AAAACCAGGAAA
1-881 FANCJ	FWD	ATAGAGATATCATGTCTTCAATGTGGTCT
	REV	ATAGAGGCCCTCAGTGATGGTGGTGATGGTG TGCATAGTCGGGGACGTCATAGGGGTACTTTTTGGAAAATTCAGCCAA
FANCJ shRNA#1 resistant primers	FWD	ACCTCTTTAAA**A**TA**T**AG**C**AC**A**TC**C**CC**C**TA**C**TTACTGGAA
	REV	TTCCAGTAA **G**TA**G**GG**G**GA**T**GT**G**CT**A**TA**T**TTTAAAGAGGT
S327A-CtIP	FWD	ACTCGAGTGTCA**GCT**CCTGTATTTGGA
	REV	TCCAAATACAGG**AGC**TGACACTCGAGT
T847A-CtIP	FWD	ATTCCACCCAAC**GCA**CCAGAGAATTTT
	REV	AAAATTCTCTGG**TGC**GTTGGGTGGAAT
