# Supplementary material for: FANCJ helicase promotes DNA end resection by facilitating CtIP recruitment to DNA double-strand breaks
Source: PLoS Genet. 2020 Apr 6;16(4):e1008701. doi: 10.1371/journal.pgen.1008701 (PMC7162537; doi:10.1371/journal.pgen.1008701)
Supplement: S4 Table — (PDF) [file pgen.1008701.s007.pdf]

**Table S4: Sequence of shRNAs used in this study**		
**shRNA**	**Sequence (5′→3′)**	**REFERENCE**
FANCJ # 1	GTACAGTACCCCACCTTAT	Litman et al. 2005
FANCJ # 2	AGCTTACCCGTCACA	Litman et al. 2005
BRCA1	GTGTGCAGCTGAGAGGCA	Gorski et al., 2010
MRE11	ACAGGAGAAGAGATCAACT	Xie et al.,2009
CtIP	GCTAAAACAGGAACGAATCTT	Yu et al., 2004
DNA2	CAGTATCTCCTCTAGCTAG	Thangavel et al., 2015
EXO1	CAAGCCTATTCTCGTATTTTT	Karanja et al., 2012
53BP1	GATACTGCCTCATCACAGT	Squatrito et al., 2012
BLM	CCGAATCTCAATGTACATAGA	Berti et al.,2013

Supplementary material references

Berti, M., Ray Chaudhuri, A., Thangavel, S., Gomathinayagam, S., Kenig, S., Vujanovic, M., Odreman, F., Glatter, T., Graziano, S., Mendoza-Maldonado, R., et al. (2013). Human RECQ1 promotes restart of replication forks reversed by DNA topoisomerase I inhibition. Nature structural & molecular biology 20, 347-354.

Gorski, J.J., James, C.R., Quinn, J.E., Stewart, G.E., Staunton, K.C., Buckley, N.E., McDyer, F.A., Kennedy, R.D., Wilson, R.H., Mullan, P.B., et al. (2010). BRCA1 transcriptionally regulates genes associated with the basal-like phenotype in breast cancer. Breast cancer research and treatment 122, 721-731.

Karanja, K.K., Cox, S.W., Duxin, J.P., Stewart, S.A., and Campbell, J.L. (2012). DNA2 and EXO1 in replication-coupled, homology-directed repair and in the interplay between HDR and the FA/BRCA network. Cell cycle 11, 3983-3996.

Litman, R., Peng, M., Jin, Z., Zhang, F., Zhang, J., Powell, S., Andreassen, P.R., and Cantor, S.B. (2005). BACH1 is critical for homologous recombination and appears to be the Fanconi anemia gene product FANCJ. Cancer cell 8, 255-265.

Squatrito, M., Vanoli, F., Schultz, N., Jasin, M., and Holland, E.C. (2012). 53BP1 is a haploinsufficient tumor suppressor and protects cells from radiation response in glioma. Cancer research 72, 5250-5260.

Thangavel, S., Berti, M., Levikova, M., Pinto, C., Gomathinayagam, S., Vujanovic, M., Zellweger, R., Moore, H., Lee, E.H., Hendrickson, E.A., et al. (2015). DNA2 drives processing and restart of reversed replication forks in human cells. The Journal of cell biology 208, 545-562.

Xie, A., Kwok, A., and Scully, R. (2009). Role of mammalian Mre11 in classical and alternative nonhomologous end joining. Nature structural & molecular biology 16, 814-818.

Yu, X., and Chen, J. (2004). DNA damage-induced cell cycle checkpoint control requires CtIP, a phosphorylation-dependent binding partner of BRCA1 C-terminal domains. Molecular and cellular biology 24, 9478-9486.
