# Supplementary material for: FANCJ helicase promotes DNA end resection by facilitating CtIP recruitment to DNA double-strand breaks
Source: PLoS Genet. 2020 Apr 6;16(4):e1008701. doi: 10.1371/journal.pgen.1008701 (PMC7162537; doi:10.1371/journal.pgen.1008701)
Supplement: S5 Table — (PDF) [file pgen.1008701.s008.pdf]

Table S5: List of Antibodies used in this study			
**Antigen name**	**Dilution**	**Source**	**Catalog #**
FANCJ	1:1000	Sigma Aldrich	B1310
BRCA1	1:200	Santa Cruz	sc-6954
BARD1	1:1000	Bethyl Laboratories	A300-263A
MRE11	1:1000	Novus Biologicals	NB100-142
CtIP	1:1000	Bethyl Laboratories	A300-488A
MLH1	1:1000	BD Biosciences	51-1327GR
RAD51	1:500	Santa Cruz	sc-8349
RPA70	1:500	Santa Cruz	sc-28304
RPA32	1:500	Santa Cruz	sc-56770
pRPA32 (S4/S8)	1:200 for IF	Bethyl Laboratories	A300-245A
BrdU	1:100 for IF	BD Biosciences	555627
γ- H2AX (pS139)	1:1000	BD Biosciences	560443
pKAP1	1:1000	Abcam	ab70369
DNA2	1:1000	Abcam	Ab96488
EXO1	1:500	Invitrogen	MA5-12262
BLM	1:500	Bethyl Laboratories	A300-110A
AceK	1:500	Cell signalling technology	#9441
53BP1	1:200	Santa Cruz	sc-22760
MCM3	1:500	Santa Cruz	sc-365616
PCNA	1:500	Santa Cruz	sc-56
α-Tubulin	1:1000	Santa Cruz	sc-5286
HA tag	1:1000	Roche	10952100
GFP tag (ChIP grade)	1:1000	Abcam	ab290
CENP-F	1: 200	Novus Biologicals	NB500-101
