# Supplementary material for: FANCJ helicase promotes DNA end resection by facilitating CtIP recruitment to DNA double-strand breaks
Source: PLoS Genet. 2020 Apr 6;16(4):e1008701. doi: 10.1371/journal.pgen.1008701 (PMC7162537; doi:10.1371/journal.pgen.1008701)
Supplement: S6 Table — (PDF) [file pgen.1008701.s009.pdf]

**Table S6: Sequences of Primers used in this study for ChIP-PCR**		
**Primer Name**	**Oligo**	**Sequence (5′→3′)**
DSB1	FWD	GATTGG CTATGGGTGTGGAC
	REV	CATCCTTGCA AACCAGTCCT
DSB2	FWD	TTCCTGCAGCCTCATTTTCT
	REV	TGATGATGCCTTTTCCCTTC
GAPDH	FWD	GCAGCCCCTTCATACCCTCACGT
	REV	GAGCCACACCATCCTAGTTGC
