# Supplementary material for: FANCJ helicase promotes DNA end resection by facilitating CtIP recruitment to DNA double-strand breaks
Source: PLoS Genet. 2020 Apr 6;16(4):e1008701. doi: 10.1371/journal.pgen.1008701 (PMC7162537; doi:10.1371/journal.pgen.1008701)
Supplement: Attachment — Submitted filename: Responses.pdf [file pgen.1008701.s011.pdf]

Responses to Editor and Reviewers (PGENETICS-D-19-01831)

Authors:

We thank all the reviewers and editor for appreciating our work and providing valuable suggestions to improve our manuscript. Most of the comments by reviewers and editor has been addressed experimentally and new data has been included in the revised version. We believe that our manuscript is greatly improved after addressing all the reviewer's comments. For editor, we have briefly indicated our responses and the detailed responses are presented for all the three reviewers' comments.

Dear Ganesh

Thank you for submitting your work to PLoS Genetics. Your manuscript was reviewed by three experts in the field and their individual comments are attached below. Moreover, I also read your manuscript with great interest and have a few specific comments, of which some relate to comments made by the other reviewers. All reviewers consider your work of potential importance and interest for this journal, but they suggest major additions and revisions to better the support the conclusions and mechanistic interpretations. I agree with their general assessment and hope that you can revise your manuscript accordingly.

The reviewers make a partially overlapping set of suggestions, and I do not think that every experimental suggestion has to be implemented in the revision. In general, I think it is important to make sure that the major conclusions are firm and corroborated by the data.

Here are my suggestions for the revised version of your manuscript:

Reviewer 1: This reviewer makes a number of excellent suggestions that would strengthen the mechanistic interpretation. While many points are listed, in my mind the major points you should focus on are:
1) Further analysis of the point mutants. See Minor Points #4, 5, 6, 7.

Authors: These are great suggestions and we have addressed all these comments experimentally and new data has been added in the revised version.

2) Additional Western blot controls (CHK2, gH2AX, RPA S4/8).

Authors: We have included these control blots for specified proteins in the revised version.

3) Survival assays would add significantly and appear possible in the AsiSI system.
RPA and RAD51 focus analysis would complement the ChIP and fractionation analysis but is not essential. I do not think that another DSB inducing agent needs to be used, as you already

show that etoposide shows the expected decrease in chromatin association of RPA70 and RAD51.

Authors: As suggested, we have performed survival experiments and data has been added in the revised version. RPA and RAD51 foci also have been measured and presented in the revised version. We have included zeocin in our analysis and new data has been presented in the revise manuscript.

About the Minor points listed: 1) Please include more discussion, but no new experiments are needed. 2) If IF is possible, this could strengthen the current interpretation. 3) This is not essential for the revision. 4-6) These are the major experimental additions. 7) Please focus on the FANCJ mutants.

Authors:

1. More discussion has been added appropriately as suggested.

2. As suggested, we have done IF for FANCJ and new data has been included.

Of the ‘Additional’, point a is a good suggestion but not needed for this revision. The remainder should pose no problem to implement.

4-6) has been addressed and included in the revised version.

7) We have included FANCJ mutants as suggested and new data is added.

Reviewer 2: The reviewer makes several specific experimental suggestions: 1) FANCJ-K1249Q-CtIP interaction. 2) Differentiation of two interaction models. Results from both experiments would add to the present data and strengthen the mechanistic model.

Authors:

1) We have included data with FANCJ-K1249Q-CtIP interaction.

2) We have addressed these two models experimentally and we have presented the new data in the revised version.

The comment about statistical significance must be addressed. The minor comments should not pose a problem.

Authors: We have taken care of statistical significance as suggested. Minor comments have been addressed.

Reviewer 3: The reviewer makes an important point whether FANCJ is directly involved in resection or via recruitment of CtIP. I agree that the phenotype of the Walker A box mutants

cannot fully settle this question. The analysis of the FANCJ-K52A mutant in the CtIP recruitment assay is an excellent suggestion. Also, the NHEJ analysis and HR analysis with the FANCJ-K1249R mutant. I think the suggested epistasis analysis with BRCA1 and 53BP1 is not needed for this manuscript. The last 4 points should not pose a problem to address with additional explanation.

Authors: These are good suggestions and we have carefully addressed these by new experiments with additional FANCJ mutants and new data has been presented in the revised version. We have performed HR and NHEJ assays for FANCJ-K1249R mutant and included in the revised version. As suggested by editor and we also believe that epistasis analysis of BRCA1 and 53BP1 is not required for the current manuscript. The remaining comments by the reviewer has been addressed.

Editor comments:
1) In the discussion, I think you have to relate your observations on resection back to your previous finding that FANCJ affects the balance between long and short tract conversion.

Authors: As suggested, we have added one new paragraph on the discussion with our current data in relation to our previous observations (Nath et al., 2017).

2) Please consult this paper (Juhasz et al. 2018 Molecular Cell) on HR in U2OS cells and consider the impact on the present and previous data.

Authors: We have included briefly about the suggested paper in our revised version.

3) I agree with reviewer 2, that the results with FANCJ KR and KA mutants needs to be presented and discussed more carefully. Has it been experimentally shown that KR binds but not hydrolyzes ATP and that KA does not bind ATP? In fact, some KA mutants bind ATP but do not lead to a conformational change that is usually accompanied by ATP binding. Moreover, in my mind the data show a differential requirement for both, KA and KR, suggesting that motor activity is needed but also conformational change after ATP binding (given the caveats above). This may suggest a 2-step model. This issue (also Rev. 2 comment on this topic) needs rewording and more discussion, but not new experiments.

Authors: These are good suggestions by reviewer and editor. We have added more discussions with FANCJ Walker motif lysine mutants with our existing and new data along with available supporting literature.

I hope these comments will be helpful for the revision and look forward to the new version of the manuscript.
My best regards
Wolf

Reviewer #1: The article titled' FANCJ helicase promotes DNA end resection by facilitating CtIP recruitment to the DNA double-strand breaks' provides insight into the role of FANCJ in promoting initial nucleolytic processing of DSBs through FANCJ interaction with BRCA1 and CtIP, components of the homologous recombination pathway of double-strand break repair. The authors claim that FANCJ has a direct role in the recruitment of CtIP through acetylation by CBP after localisation to DSBs in a BRCA1 dependent manner through phosphorylation by CDK. Furthermore, the authors claim that the helicase activity of FANCJ is also required for DNA resection. These claims are generally not very well supported and require significant amount of experimental work to solidify the hypothesis.

Authors: We appreciate all the comments and concerns by this reviewer. We have addressed all the concerns experimentally and the new data has been included in the revised version.

Major comments:

The authors provide insight into the impact of FANCJ on ssDNA generation through resection primarily using the ER-AsiSi system. While the author's provide an initial additional read-out for ssDNA through the analysis of BRDU foci (unusually high in control given the number of DSBs generated by tamoxifen-dependent induction of ER-AsiSi), it is essential that the authors conduct similar analysis to complement the findings associated with separation of function point mutants identified.

Authors: As suggested, we have validated our data obtained from ER-AsiSi system with BrdU foci for all the FANCJ mutants. Moreover, we have reperformed all the BrdU experiments by including more washes to eliminate non-specific signals and captured the BrdU foci in a new microscope. The new data has been presented in the revised version (Fig. 1B, 1C, S8B, S8C, S9B, S9C, S10B, S10C, S14B and S14C).

In addition, (i) the inclusion of additional controls in AsiSi DSB resection efficiency analysis is essential such as (ii) western blots showing level of RPA S4/8, CHK2 and gH2AX for FANCJ-deficient, CtiP-deficient (internal control) and the doubles. These should be done in response to Tamoxifen treatment as well as to a DSB inducing agent, such as zeocin.

Authors: As suggested, we have performed western blots for all the suggested proteins and new data has been presented in Fig. S3A and S3B. We have included zeocin in our experiments as suggested.

It is somehow surprising the level of foci shown in Figure 1B and supplementary figure 2a upon tamoxifen treatment (BrDU and pRPA2 S4/S8, gH2AX) given that such treatment should generate relatively small number of DSBs – do the authors see the same pattern of response upon zeocin treatment?) – this would be an essential analysis to include (also for the separation of function mutants).

Authors: This concern has been addressed with new experiments and new data has been presented in Fig. S2.

Since defect in resection would result in a defective RPA and RAD51 foci formation these should be analysed as well (upon tamoxifen as well as zeocin treatment).

Authors: As suggested, we have analyzed RPA and RAD51 foci and data is presented in Fig. S4 and S5.

Finally, survival assays for FANCJ and the separation of function mutants should be included in response to tamoxifen as well as zeocin (or another DSB inducing agent).

Authors: Survival assays have been performed for all the FANCJ mutants for AsiSI induced breaks and the data has been presented in Fig. S8D, S9D, S11C and S14D. End resection phenotype for FANCJ has been confirmed by tamoxifen, etoposide and zeocin. We believe and as indicated by editor, extending the survival assays for other agents would be redundant.

Minor points to address:

1. Figure 2A shows that shMRE11 results in a significant defect in FANCJ recruitment to DSBs which may support previous reports. However, an explanation for this is not addressed throughout the article. Why does MRE11 deficiency affect FANCJ recruitment- what is the mechanism and evidence for this in the authors hands or from the literature? This should be included in text.

Authors: As suggested, we have included more discussion on MRE11 depletion affecting the FANCJ loading to DSBs. MRE11 interacts with C-terminus of FANCJ and deletion of this region abolishes MRE11 interaction (Fig. 3B). Since MRE11 localizes to the sites of DSBs as one of the early proteins, it will facilitate FANCJ recruitment by its physical interaction. A study by Brosh group also had a similar observation (Suhasini et al., 2013, MCB) and we have cited this reference in the revised version.

2. Immunofluorescence microscopy for FANCJ recruitment to AsiSi breaks should complement ChIP and resection analysis in Figure 2 (see also major comments above).

Authors: As suggested, we have included this data in Fig. S6 in the revised version.

3. Figure 2B and C: BARD1/ BRCA1-FANCJ interaction appears to be upregulated after DSB induction. Given that they form a stable complex and together are shown to counteract 53bp1 function to promote DNA resection (Densham et al., 2016. Nat. Struc. Biol), including BARD1 and BRCA1 in ChIP analysis (Figure 2C) to determine whether FANCJ depletion affects their recruitment is advised.

Authors: 53BP1 and BRCA1 are upstream players in channelizing the DSBs for either HR and NHEJ mediated repair. Our previous study (Nath et al., 2017, NAR) as well as study from Sharon Cantor group (Litman et al., 2005, Cancer Cell) shows that FANCJ participates in HR mediated DSB repair. Present manuscript clearly shows the FANCJ role in end resection and thereby affects HR. We believe and as indicated by editor, analyzing the BRCA1 and BARD1 would be beyond the scope of current study.

4. Resection analysis in Figure 4C and 5C should include the CΔ-FANCJ mutant as comparison to separate function to BRCA1 and CtIP, this will enable insight into relative contribution to DNA resection based on BRCA1/CtIP- FANCJ interaction vs interaction with additional DNA resection factors (specifically MRE11). This will underline the role of the identified point mutations and FANCJ in the wider mechanism of HR.

Authors: As suggested, we have included CΔ-FANCJ mutant in our studies with FANCJ phosphorylation and acetylation mutants to strengthen our findings. The new data has been presented in Fig. 4C, 5C, S9A and S10A.

5. Additional experimental read-outs for resection defects are required for all separation of function mutants analysed: FANCJ S990A/E, K1249R/Q, CtIP-T847A, FANCJ K52A/R. BRDU foci analysis is proposed to support resection efficiency assays.

Authors: We have performed new experiments measuring BrdU foci for all the FANCJ mutants and the new data has been included in Fig. 1B, 1C, S8B, S8C, S9B, S9C, S10B, S10C, S14B and S14C.

6. Figure 7 C displays resection efficiency after depletion of CtIP followed by rescue analysis using mutants. Resection analysis should include shFANCJ and shFANCJ-K1249R to show epistasis or relative contribution of complexes to resection efficiency.

Authors: We have already performed these studies with and without depletion of FANCJ (Fig. 8C in the revised version). Depletion of either FANCJ or CtIP or co-depletion of both shows the same effect, indicating that FANCJ and CtIP acts in the same pathway to regulate end resection. This is further supported with our data presented in Fig. 2D, Fig. S7B and Fig. 2E.

7. Figure 8 C: Resection analysis should include FANCJ-S990A and K1249R separation of function mutants or CtIP or BRCA1 shRNA treatments. If the interactions with CtIP or BRCA1 is epistasic to the helicase activity. Authors may also include K52A + K1249R FANCJ double

point mutants as additional control to demonstrate epistasis. This will solidify the mechanism described.

<u>Authors:</u> As suggested, we have included double FANCJ mutants in our studies and new data has been presented in Fig. 9D and S15.

Additional:
a. Use of CDK or CBP inhibitors followed by analysis of interactions with CtIP will reinforce the claim of their direct role in regulating FANCJ.

<u>Authors:</u> Although it is a good suggestion, we and editor believe that it is not required for the present study.

b. Figure S3C should be included in Figure 2 as additional panel.

<u>Authors:</u> We have moved this data.

c. Figure S4A should be included in Figure 3 as additional panel.

<u>Authors:</u> We have shifted this data.

Thorough checking of sentence structure and grammar is required throughout article.

<u>Authors:</u> We agree that there were few minor errors which we have taken care to the best of our knowledge in the revised version.

Reviewer #2: In this study the authors report on the interaction of FANCJ with CtIP that they found to be required for the recruitment of CtIP to DNA double-strand breaks (DSBs). They further show that the interaction with CtIP is dependent on FANCJ acetylation at K1249, which in turn requires S990 phosphorylation. Overall, the experiments in this paper are carefully conducted and the results are clearly presented.

<u>Authors:</u> This reviewer appreciates our work and provide good suggestions for further improvement.

Major comments:

– The authors show that the interaction between FANCJ and CtIP depends on DSBs (Figure 2B), but do not show whether this is due to acetylation of FANCJ in response to DSBs. If so, FANCJ K1249Q should be able to interact with CtIP also in the absence of DSBs. The authors should test this.

Authors: As suggested, we have studied FANCJ interaction with CtIP in the absence of damage using K1249Q mutant and we find that this mutant interacts with CtIP. This data has been added in Fig. S13A.

– The authors show that phosphorylation of FANCJ at S990 is required for acetylation at K1249 and that both FANCJ S990A and K1249R are unable to interact with CtIP. In my view there are two possible interaction modes between FANCJ and CtIP. First, phosphorylation of S990 is required for K1249 acetylation (for example through recruitment of CBP or another acetyltransferase), but CtIP physically only binds to acetylated K1249; or second, phosphorylation of S990 is required for K1249 acetylation and CtIP binds to both phosphorylated S990 and acetylated K1249. I would encourage the authors to utilise a double mutant (S990A/K1249Q) and do CtIP interaction experiments (similar as in Figure 6A) to distinguish between these two possibilities.

Authors: This is an interesting hypothesis by the reviewer. As suggested, we have carried out our studies with indicated FANCJ double mutants. Our new data clearly suggests the first model, where in FANCJ exclusively interacts with acetylated FANCJ. This data is presented in Fig. S12.

– It is interesting that the authors see different phenotypes with FANCJ K52A and K52R. However, they should be more careful with their wording (results section and abstract). Given that they see a partial rescue with K52R, it would appear that ATP binding (as opposed to ATP hydrolysis and helicase activity) is sufficient to partially restore resection.

Authors: As suggested, we have included more discussions with the data from FANCJ K52A and K52R mutants. Our extended study with FANCJ double mutants in combination with Walker motif lysine mutants suggest that in addition to ATP binding induced conformational changes, its hydrolysis is also important for FANCJ mediated proficient end resection. Moreover, with our studies, it is clear that FANCJ in addition to its scaffolding role in CtIP recruitment, its motor activity is also critical for end resection. These discussions have been included in the revised version and indicated in the abstract.

– Figure 8C and S7A: Statistical significance should be calculated between “shFANCJ + WT” and “shFANCJ + K52A” or “shFANCJ + K52R” and not between “shFANCJ + K52A” and “shFANCJ + K52R”.

Authors: We have taken care of this and presented in Fig. 9D and S14A.

Minor comments:

– Introduction, line 90: It should read “maintenance of microsatellite stability”

Authors: We have corrected

– Figure 1D: The authors should mention somewhere why they include knock-down of DNA2 and 53BP1.

Authors: We have elaborated our data with DNA2 and 53BP1 (Fig. 1D) in results section with references.

– Results, line 198: It should read "FANCJ K1249R was defective…" not " FANCJ K1249R expressing cells…"

Authors: We have corrected this mistake.

– Results, lane 199-201: Since the authors show before that CtIP is not required for recruitment of FANCJ to DSBs, it is not so surprising that FANCJ K1249R can still localise to DSBs. Please rephrase.

Authors: We have modified this as suggested.

– Table S4: Change title (since it contains not only SDM primers) and highlight all nucleotide changes for SDM primers.

Authors: We have changed.

Reviewer #3: Previous studies have shown a decreased frequency of homologous recombination (HR) in the absence of FANCJ. In this study, the authors investigate the role of FANCJ in the earliest step of HR, DNA end resection. They find reduced generation of single-stranded DNA (ssDNA) after induction of AsiSI endonuclease and show lower accumulation of CtIP at DSBs in FANCJ-depleted cells. Furthermore, they find that CtIP recruitment/retention at DSBs requires phosphorylation and acetylation of FANCJ, and provide evidence that the CtIP-FANCJ interaction is independent of BRCA1, a known interaction partner of both CtIP and FANCJ.

The findings presented support a role for FANCJ in recruitment of CtIP to DSBs to facilitate end resection. It is unclear from the data whether FANCJ plays a direct role in end resection or if its primary function is in recruitment of CtIP.

Authors: This is a good suggestion/concern. We have addressed this more carefully with additional FANCJ mutants. The new data presented in Fig. 9D and S15, shows that FANCJ in addition to its scaffolding role in CtIP recruitment also requires its helicase activity for proficient end resection. We have added more discussion in the revised version.

The end resection defect of the FANCJ-K52A mutant would appear to suggest a catalytic role, but it is not obvious why MRN-CtIP would require a helicase to initiate end resection. Is CtIP recruited to DSBs in cells expressing FANCJ-K52A?

Authors: It is a good suggestion; we have analyzed the CtIP recruitment in FANCJ K52A mutant and find that this mutant is proficient in recruiting CtIP to DSBs but defective for end resection. The new data has been added in Fig. 9C. This further suggests that in addition to FANCJ mediated CtIP loading its motor activity is also critical for end resection. These points have been clearly discussed in the revised manuscript.

If FANCJ is acting at an early step it would be expected to shift the balance from HR to NHEJ. I suggest the authors measure NHEJ efficiency in FANCJ depleted cells. I also recommend measuring HR in cells expressing the FANCJ-K1249R mutant.

Authors: As suggested, we have performed HR and NHEJ assays with K1249R FANCJ mutant and we find an increase in NHEJ and reduction in HR. This new data has been added in Fig. S11A and S11B.

To confirm that FANCJ functions independently of BRCA1 in end resection, I suggest measuring end resection in sh53BP1 shFANCJ cells and comparing to sh53BP1 shBRCA1 cells.

Authors: We believe and as agreed by editor, addressing this comment is not required for the current work.

Most of the assays rely on induction of DSBs by AsiSI. Is there equivalent efficiency of AsiSI cleavage at DSB 1 and 2 in the all of the cell lines expressing shRNAs? Were the values of ssDNA shown for the end resection assay normalized to the % AsiSI cleavage at each site?

Authors: AsiSI system was developed by Gaelle Legube and Tanya Paull's group and they find less no. of breaks at DSB2 site (Zhou, et al., 2014, NAR). In all our assays we have normalized ssDNA generation to % AsiSI cleavage at each site.

Most of the resection assays show a single time point, 4 h, after DSB induction. Were other time points analyzed? Does resection in FANCJ-depleted cells increase at later time points? Why were ChIP assays done 2 h after 4-OHT treatment? Later time points should also be used to measure RAD51 association with DSBs.

Authors: Our initial experiments carried out for optimization showed that maximum recruitment of end resection factors occurs at 2h post 4-OHT treatment and maximum resection was

observed at 4hrs. RAD51 recruitment gradually reduces after 4h. In fact, a recent study also shows a similar observation (Tripathi et al., 2018, Nat. Comm).

The methods for transfection with shRNA constructs are not explained in enough detail.

Authors: We have added more details with transfections.

How long after transfection were cells treated with 4-OHT to induce AsiSI? Do cells remain arrested in G2 after transfection with shRNAs and induction of AsiSI? At what time point were Western blots performed to assess knockdown efficiency?

Authors: For single shRNA constructs, after 40h of transfection, we have added 4-OHT to induce breaks. For shRNA resistant constructs, we have added 4-OHT after 16hrs of second transfection. In BrdU assay, BrdU positive cells were specifically analyzed in S/G2 phase cells using CENP-F as a marker. For knockdown analysis, we have done western blotting after 48h of transfection with shRNA constructs. These details have been added in the revised version.

Line 150: I don't think the authors can conclude direct interaction based on IP results. The authors would need to demonstrate interaction between the purified proteins to conclude that it is direct.

Authors: In our studies we find that interactions of FANCJ with CtIP or BRCA1 is dependent on posttranslational modifications such as acetylation and phosphorylation. Hence, understanding their direct interactions with purified proteins would be difficult.
