# Supplementary material for: FANCJ helicase promotes DNA end resection by facilitating CtIP recruitment to DNA double-strand breaks
Source: PLoS Genet. 2020 Apr 6;16(4):e1008701. doi: 10.1371/journal.pgen.1008701 (PMC7162537; doi:10.1371/journal.pgen.1008701)
Supplement: Attachment — Submitted filename: Responses to Reviewers.pdf [file pgen.1008701.s012.pdf]

Responses to Reviewers (PGENETICS-D-19-01831)

Authors:

We thank all the reviewers and editor for appreciating our work. Minor suggestions/comments were also very useful for us to modify the manuscript and make it to the best form. Please find here below detailed responses.

Reviewer #1: The manuscript has been now substantially improved and I would like to congratulate the authors on this very nice pice of work.

Minor comments:

1. Canonical image for Fig S2A shCtIp pRPAS4/S8 stain does not support graphical representation in Fig S2B.

Authors: As suggested, we have replaced this image with a new one (Fig. 1C).

2. shCtIP canonical image for RAD51 staining (Fig S4C) does not complement graph trend (Fig S4D).

Authors: We have replaced it with a new image (Fig. S2D).

Reviewer #2: The authors did a very good job in addressing the reviewers' comments with many new experiments. I think they report important findings that will be of great interest to the readership of PLOS Genetics.

However, I still have a few remaining criticisms:

– I still think that the issue KA/KR is not properly addressed. For example, in the abstract, the authors write "its helicase activity is crucial for promoting end resection". Given Figure 9D where the authors see a partial restoration of ssDNA with the KR (but not the KA) variant, this is not correct. They should instead use the term "intact ATP binding domain" or "ATP binding". Same problem in the results and discussion sections.

Authors: As suggested, we have modified our writing in the abstract, results and discussion.

– I think the manuscript requires careful copy-editing. There are issues with grammar, hyphenation, articles etc.

Authors: We agree with the reviewer. There were still some few minor errors in the manuscript which we have corrected by taking help of **"Grammarly"** online free software. The marked version of the revised manuscript has been submitted for your reference.

– I also think that the flow of the paper could be improved – ironically, I think this problem comes partially from the wealth of new data that were added. It would certainly help to add more sub-headings to the results section and to try and integrate the SI data in a better way.

Authors: This is a great suggestion by this reviewer. Indeed, we also realized most of our important data was still in the supplementary material. As suggested, we have moved most of our supplemental data to main figures. We also added two new subheadings in the results section. With these changes, the manuscript looks more appealing.

Minor:

– Not all SI figures come in the right order in the text.

Authors: Since we have moved most of the SI figures to main figures, the manuscript flow is good.

– Figure S6A/B: I would swap red/green in the graph to be consistent.

Authors: For 4-OHT experiments the γH2AX is denoted by FITC signal, whereas for zeocin this is with TRITC signals. Hence, we have retained the data as it is. In the revised manuscript, we have separated the data with 4-OHT and zeocin to main and supplementary figures, respectively. Hence the problem has been solved.

– Figures S12: Please align MRE11 blot.

Authors: We have aligned this as suggested.
